# Co-Inhibition of Androgen Receptor and PARP as a Novel Treatment Paradigm in Prostate Cancer—Where Are We Now?

**DOI:** 10.3390/cancers14030801

**Published:** 2022-02-04

**Authors:** Arpit Rao, Nagaishwarya Moka, Daniel A. Hamstra, Charles J. Ryan

**Affiliations:** 1 Division of Hematology and Oncology, Dan L. Duncan Comprehensive Cancer Center, Baylor College of Medicine, Houston, TX 77030, USA; 2 Division of Hematology and Oncology, Baylor College of Medicine, Houston, TX 77030, USA; nagaishwarya.moka@bcm.edu; 3 Department of Radiation Oncology, Baylor College of Medicine, Houston, TX 77030, USA; daniel.hamstra@bcm.edu; 4 Division of Hematology, Oncology and Transplantation, Masonic Comprehensive Cancer Center, University of Minnesota, Minneapolis, MN 55455, USA; ryanc@umn.edu

**Keywords:** synthetic lethality, novel therapy, metastatic prostate cancer, advanced prostate cancer, metastatic castration-resistant prostate cancer, PARP inhibitor combination

## Abstract

**Simple Summary:**

The past decade has seen the development and regulatory approval of a large number of new treatment options for advanced prostate cancer. Despite this, the two most used treatments—abiraterone and enzalutamide—have not been surpassed in efficacy, and eventually, all patients develop resistance to these treatments. Co-inhibition of androgen receptor (AR) and PARP proteins results in cancer cells’ inability to repair DNA. This combination approach has shown impressive early efficacy signals in preclinical and clinical settings and appears to be poised for a breakthrough. We review the science, data from clinical trials, and the future directions for this approach in this article.

**Abstract:**

Metastatic prostate cancer remains lethal with a 5-year survival rate of about 30%, indicating the need for better treatment options. Novel antiandrogens (NAA)—enzalutamide and abiraterone—have been the mainstay of treatment for advanced disease since 2011. In patients who progress on the first NAA, responses to the second NAA are infrequent (25–30%) and short-lasting (median PFS ~3 months). With the growing adoption of NAA therapy in pre-metastatic castration-resistant settings, finding better treatment options for first-line mCRPC has become an urgent clinical need. The regulatory approval of two PARP inhibitors in 2020—rucaparib and olaparib—has provided the first targeted therapy option for patients harboring defects in selected DNA damage response and repair (DDR) pathway genes. However, a growing body of preclinical and clinical data shows that co-inhibition of AR and PARP induces synthetic lethality and could be a promising therapy for patients without any DDR alterations. In this review article, we will investigate the limitations of NAA monotherapy, the mechanistic rationale for synthetic lethality induced by co-inhibition of AR and PARP, the clinical data that have led to the global development of a number of these AR and PARP combination therapies, and how this may impact patient care in the next 2–10 years.

## 1. Introduction

Advances in prostate cancer treatments have reduced the mortality rate by 52% in the last 2 decades from 39.3 per 100,000 cases in 1993 to 18.8 per 100,000 cases in 2017 [1]. Yet, prostate cancer remains the second-leading cause of cancer-related mortality in men, with a dismal 5-year survival rate of 30.5% for patients with metastatic disease [2,3]. A majority of prostate cancer patients are still receiving primarily or exclusively androgen-receptor (AR)-directed therapies such as androgen deprivation therapy (ADT) or novel antiandrogens including enzalutamide and abiraterone [4]. Subsequent to their initial regulatory approval for post-chemotherapy metastatic castration-resistant prostate cancer (mCRPC) setting, these two agents, in particular, have found success in prolonging overall survival in earlier disease settings, including metastatic hormone-sensitive prostate cancer (mHSPC). It is well known that subsequent NAA therapy only works in a minority of patients, and the responses are short-lasting (median PFS ~3 months for second NAA in mCRPC) [5,6,7,8,9]. With a growing proportion of patients now receiving their first NAA therapy prior to developing castration-resistant disease, an urgent evaluation of non-NAA monotherapy approaches is warranted.

In this article, we will review the scientific rationale, preclinical and early clinical data, and future possibilities of one such approach that combines AR and poly (ADP-ribose) polymerase (PARP) inhibition to induce synthetic lethality in prostate cancer cells.

## 2. Limitations of NAA Monotherapy in Advanced Prostate Cancer

Abiraterone and enzalutamide were the first two NAA therapies approved for mCRPC [4]. Abiraterone inhibits androgen biosynthesis via inhibition of the cytochrome P450 17α-hydroxylase/C17,20-lyase enzyme intratumorally and in the adrenal glands. Enzalutamide is a potent, direct AR-antagonist that competitively inhibits androgen binding to AR and blocks its function as a transcription factor intratumorally.

Abiraterone and enzalutamide are practically indifferentiable in efficacy as first-line agents in mCRPC, as suggested by a number of head-to-head studies (Table 1). In non-mCRPC settings, the other NAA therapies that have received regulatory approval—apalutamide and darolutamide—also have a similar efficacy profile as enzalutamide. In general, the selection of an NAA in these settings is primarily guided by physician and payor preferences and adverse effect profiles [4].

In earlier disease settings, such as metastatic hormone-sensitive (mHSPC) or non-metastatic castration-resistant (nmCRPC) prostate cancer, where the dominant disease biology is AR-dependent [13,14,15,16,17], and patients tend to have a long duration of therapeutic exposure [18,19,20,21,22], the requirement for combinatorial approaches remains limited to a subset of patients who have aggressive disease biology (e.g., combination cytotoxic chemotherapy is routinely used for de novo neuroendocrine carcinoma that has spread beyond the prostate) or other indications for treatment intensification (e.g., patient preference). However, the dramatic increase in usage of NAA monotherapy in non-mCRPC settings since 2017 has increased the urgency of exploring non-AR-driven or combinatorial approaches for patients who develop mCRPC after treatment with an NAA.

Clinical trajectory prior to mCRPC is important even for patients who did not receive a prior NAA therapy. This is because the clinical trials that led to the approval of abiraterone (COU-AA-302) [23] and enzalutamide (PREVAIL) [19] in pre-chemotherapy mCRPC only enrolled patients with asymptomatic or mildly symptomatic disease and excluded those needing opiate analgesia or radiation for prostate cancer prior to enrollment. Furthermore, the PREVAIL trial had 12% patients with visceral (lung and/or liver) metastatic disease; the COU-AA-302 trial excluded such patients entirely. While such patients are routinely treated with NAA therapy in contemporary clinical practice, at least in our clinical experience, the responses tend to be less durable, likely due to the underlying disease biology [24]. There are no combination therapies currently approved for first-line mCRPC, and for the two groups of patients outlined above, treatment intensification may yield the greatest benefit. 

A031201, a randomized phase 3 trial of 1311 men with mCRPC, evaluated the combination of enzalutamide and abiraterone (enz+abi) vs. enzalutamide alone (enz) [25]. The trial failed to show an OS benefit with enz+abi compared to enz (33.6 vs. 32.7 months, respectively; *p* = 0.19). PSA declines were comparable in the two arms as well, but there was a trend towards improved rPFS with enz+abi compared to enz (25.2 vs. 20.7 months, respectively; *p* = 0.02). The combination had considerably higher adverse event (AE) rates, with grade 3–5 AE (all attributions) of 68.8% in enz+abi vs. 55.6% in enz arms. Key AEs that were more common with enz+abi included fatigue, hypertension, and atrial fibrillation.

Adding a second NAA to patients who are starting to progress on the first NAA has not been shown to improve outcomes either. In the PLATO trial, 509 men with mCRPC were treated with enzalutamide, and those with no PSA increase at weeks 13 and 21 were then randomized to either enz+abi or enz alone upon PSA progression (≥25% increase and ≥2 ng/mL above nadir) [26]. Median PFS (radiographic or unequivocal clinical progression) was 5.7 months in the enz+abi arm and 5.6 months in the enz arm (HR = 0.83; 95% CI, 0.61 to 1.12; *p* = 0.22). There were no differences between groups in objective response rates, the rates of pain progression, or time to the first use of subsequent antineoplastic therapy. AE rates were higher in the enz+abi arm with grade ≥3 AEs of 45% in enz+abi vs. 37% in enz arm. The most common AEs in the enz+abi arm were hypertension (10% vs. 2% in enz arm), increased ALT (6% vs. 2%), and increased AST (2% vs. 0%).

### Sequencing Considerations in Second and Subsequent Lines of Therapy

Several prospective and retrospective studies have evaluated the optimal sequencing of abiraterone and enzalutamide [10,11,12,27,28], including a randomized, phase 2, crossover trial of 202 men with mCRPC assigned in 1:1 fashion to either (1) abiraterone acetate plus prednisone until PSA progression followed by enzalutamide or (II) enzalutamide until PSA progression followed by abiraterone [10]. Time to second PSA progression was longer in the abiraterone-first group than the enzalutamide-first group (median 19.3 vs. 15.2 months; hazard ratio (HR) 0.66, 95% CI: 0.45–0.97, *p* = 0.036), at a median follow-up of 22.8 months. Additionally, a greater proportion of patients had PSA response to second-line enzalutamide therapy (36%) than abiraterone therapy (4%; χ^2^ *p* < 0.0001). 

Based on these data (Table 1), our preferred approach is to give NAA-naïve patients abiraterone before enzalutamide in the mCRPC setting. 

Given the differences in mechanisms of action, it is possible that these findings may be extrapolated to earlier disease states. However, the long-term follow-up data from the SPARTAN phase 3 study of apalutamide and ADT vs. placebo and ADT in nmCRPC showed that response to subsequent NAA in first-line mCRPC could be longer than anticipated [20,29]. In this study, patients who progressed to metastatic disease were offered treatment with abiraterone, and >70% of patients in either arm received study-sponsored abiraterone as the first subsequent therapy after progression. The primary endpoint of this study was metastasis-free survival (MFS). In the apalutamide arm, the median post-metastasis survival was 33.4 months, which is similar to the previously reported median OS of 34.7 mo for the abiraterone arm in the COU-AA-302 study in first-line mCRPC [23]. In addition, the difference between median MFS and PFS on the first subsequent therapy (PFS2) was 15.1 months, which is close to the median rPFS of 16.5 months reported in COU-AA-302.

## 3. DNA Repair and HRR Gene Alterations in Prostate Cancer

Eukaryotic cells employ several DNA damage response and repair (DDR) pathways to maintain genomic integrity and stability. DDR pathways are generally classified into single-stranded DNA (ssDNA) repair and double-stranded DNA (dsDNA) repair pathways based on their primary mechanism of action. DDR is a complex, three-phase process that involves DNA damage detection, accumulation of repair factors, and DNA-damage repair. Once DNA damage is detected, cell cycle arrest and DNA repair machinery are initiated. If the cell incurs substantial, unrepaired DNA damage, apoptosis is initiated [30,31,32]. Any combination of treatments that overwhelm DDR machinery by causing excessive DNA damage, impaired DNA repair, or both, could provide a valuable therapeutic option. 

The PARP family of enzymes (mostly PARP1, 2, and 3) play an important role in DNA repair [33,34,35,36]. PARP1 binds to ssDNA and dsDNA breaks, which stimulates its catalytic activity by 500-fold. PARP1 then cleaves nicotinamide adenine dinucleotide (NAD+) and transfers the resulting ADP-ribose onto itself or other target proteins in a reaction called polyADP-ribosylation (PARylation). This post-translational modification auto-activates PARP and other DNA-repair enzymes.

PARP1 is especially important for ssDNA repair due to its ability to recruit XRCC1, a core repair protein that acts as a scaffold for DNA-repair machinery in the base excision repair pathway [36]. PARP1 is also recruited to dsDNA damage sites. The rapid PAR-dependent recruitment of mitotic recombination 11 (MRE11) [37] and ataxia telangiectasia-mutated (ATM) [38,39] hints at PARP1’s important role in the homologous recombination repair (HRR) pathway. PARP1 facilitates recruitment of the chromatin remodeler CHD2 to dsDNA breaks, which then recruits the core components of the non-homologous end rejoining (NHEJ) pathway [40]. 

Synthetic lethality is a phenomenon where the simultaneous loss of function of two genes results in cell death, but the loss of function of only one gene does not [41]. Synthetic lethality between PARP inhibition and BRCA mutation or depletion was first observed in 2005 based on the hypothesis that inhibition of PARP1 would result in the collapse of the replication fork and a cell with impaired HRR-dependent repair of these forks would not remain viable [42,43].

Germline or somatic HRR aberrations occur in a quarter of mCRPC patients [13,15,16,44,45]. BRCA2 (12–18%), ATM (3–6%), CHEK2 (2–5%), and BRCA1 (< 2%) are the most commonly affected HRR genes in prostate cancer. Germline aberrations represent roughly half of the cases of HRR mCRPC, with BRCA2 (5.3%) followed by ATM, CHEK2, and BRCA1 (1.9, 1.6, and 0.9%, respectively) as the most frequent affected genes [46].

## 4. PARP Inhibitor Monotherapy in Prostate Cancer

The past decade has seen several therapeutic breakthroughs for prostate cancer, including the approval of two PARP inhibitors—rucaparib and olaparib—for mCRPC patients harboring germline or somatic aberrations in DDR genes such as *BRCA1* and *BRCA2* [4]. These agents were the first true targeted therapies to be approved in prostate cancer as a response requires the presence of DDR aberrations. 

Rucaparib was the first PARP inhibitor approved by the U.S. Food and Drug Administration (FDA) in mCRPC harboring *BRCA1/2* mutations after treatment with at least one NAA and one taxane chemotherapy [47]. This accelerated approval was based on the TRITON2 multicenter, single-arm phase 2 clinical trial [48]. A total of 115 patients with *BRCA1* (n = 13) or *BRCA2* (n = 102) received rucaparib 600 mg orally twice daily. Sixty-two (53.9%) patients had measurable disease per protocol-specified criteria. The confirmed ORR (primary endpoint) was 43.5% (27/62; 95% CI, 31.0–56.7%) including complete response rate of 11.2% (7/62). Most (88.7%) patients had the best response of stable disease or better. Median radiographic PFS (rPFS) was 9.0 months (95% CI, 8.3–13.5 months).

Olaparib received full FDA approval shortly after rucaparib. However, eligibility for olaparib is much broader for two reasons—the regulatory label only requires treatment with one prior NAA (no taxane requirement) and patients with one of 15 HRR gene alterations (*BRCA1*, *BRCA2*, *ATM*, *BRIP1*, *BARD1*, *CDK12*, *CHEK1*, *CHEK2*, *FANCL*, *PALB2*, *PPP2R2A*, *RAD51B*, *RAD51C*, *RAD51D*, and *RAD54L*) are eligible [49].

This approval was based on the PROFOUND randomized phase 3 trial in which mCRPC patients previously treated with an NAA and harboring any of these 15 HRR gene mutations were randomized in 2:1 fashion to olaparib 300 mg orally twice daily or physician’s choice of standard-of-care enzalutamide or abiraterone (control) [45]. The median rPFS (primary endpoint) was significantly longer in the olaparib arm than in the control arm (7.4 vs. 3.6 months; HR 0.34; 95% confidence interval (CI), 0.25 to 0.47; *p* < 0.001). The confirmed ORR among evaluable was 33% (28/84 patients) in the olaparib arm and 2% (1/43 patients) in the control arm (odds ratio 20.86; 95% CI, 4.18 to 379.18; *p* < 0.001). The median overall survival (OS) favored olaparib at the interim analysis (18.5 vs. 15.1 months in control arm; HR 0.64; 95% CI, 0.43 to 0.97; *p* = 0.02)

Niraparib received FDA’s breakthrough therapy designation in 2019 based on data from the GALAHAD, a phase II, multicenter, open-label clinical trial of niraparib monotherapy in the treatment of adult patients with HRR-aberrant mCRPC who received treatment with next-generation androgen-receptor targeting therapies and docetaxel [50]. This agent is not FDA approved in prostate cancer. Talazoparib is another PARP inhibitor in advanced stages of clinical development but is not FDA approved for prostate cancer [51].

Only 15–25% of patients who harbor these DDR aberrations as outlined above and are, in theory, eligible for PARP inhibitor monotherapy. In clinical practice, barriers such as the lack of sufficient tissue biopsy material for next-generation sequencing using a companion diagnostic assay render some of these patients ineligible for PARP inhibitor monotherapy. Thus, only a small proportion of prostate cancer patients derive the benefits of PARP inhibitor monotherapy.

## 5. Synthetic Lethality by Co-Inhibition of AR and PARP

For decades, ADT combined with radiotherapy has been shown to be superior to radiation therapy alone and has been the standard of care in localized and locally advanced prostate cancer [52,53,54]. These therapies were initially combined based on clinical availability, but an interrogation of the underlying biology has shed light on the role of AR in DDR since radiation therapy’s primary mechanism of action is induction of dsDNA breaks [55,56,57]. This does not seem to be a merely additive effect since ADT combined with radical prostatectomy has not yielded any meaningful improvement in outcomes for these cohorts of patients [58,59,60,61,62,63].

**Figure 1:**
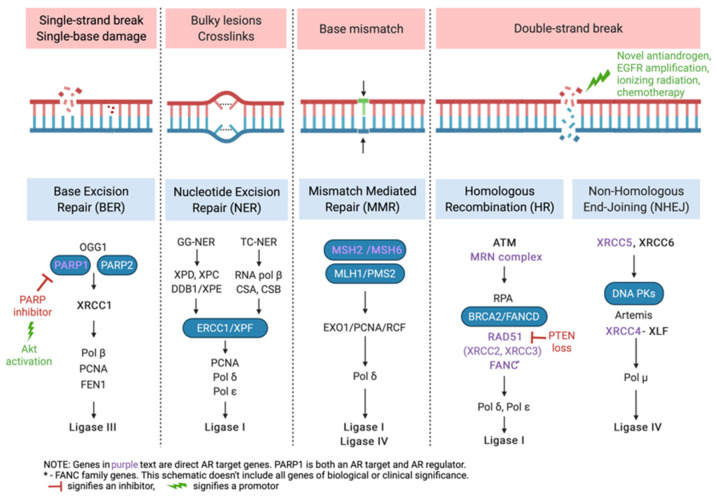
Synthesizing lethality—the interplay between androgen receptor-regulated genes and PARP family of proteins in DNA damage response [65].

Preclinical experiments have shown that NAA exposure downregulates DNA repair gene expression in CRPC xenografts, that DNA repair genes are direct AR targets, and that the synergy between ionizing radiation and AR inhibition is mediated by DDR pathways (Figure 1) [64].

Subsequent experiments have shown that AR activation results in the expression of DNA damage repair genes, including PRKDC, encoding DNA-dependent protein kinase catalytic subunit (DNA-PKcs), an essential protein necessary for nonhomologous end-joining (NHEJ) repair of double-stranded DNA (dsDNA) break [66,67,68,69]. In addition, treatment with androgens results in the upregulation of XRCC2 and XRCC3, two genes important for homologous recombination (HR) [70,71]. NAA treatment results in decreased DNA repair in cells and increased levels of dsDNA breaks (Figure 1). This opened an opportunity to combine NAA with PARP inhibitors, given the latter’s role in ssDNA repair pathways.

### 5.1. Summary of Clinical Trial Data on Synthetic Lethality with NAA and PARP Inhibitors

The first clinical trial to demonstrate an improvement in outcomes with NAA and PARP co-inhibition was a randomized, placebo-controlled, phase II trial of abiraterone with olaparib (abi+ola) vs. abiraterone plus placebo (abi). In this multicenter trial, 142 men with mCRPC previously treated with docetaxel but not an NAA were randomized 1:1 to abi+ola or abi [72]. No a priori stratification or treatment selection based on HRR aberration status was undertaken, but all patients underwent next-generation sequencing for assessment of HRR mutation status. In the intent-to-treat (unselected) population, abi+ola combination showed a superior rPFS (median 13.8 months) compared with abi alone (median 8.2 months), translating into a 35% improvement in the risk of radiographic progression or death (HR 0.65, 95% CI 0.44–0.97, *p* = 0.034). 

Notably, the criteria for designating a patient as an HRR wild-type were to have no detectable HRR mutation and a valid tumor NGS test result from the archival tissue specimen. Subsequent analysis of patients with HRR partially characterized status (61%, or 86/142) found that 93% (80/86) of these patients met the definition of HRR wild-type on plasma circulating tumor DNA or germline testing. Thus, a majority of patients in this study (81%; 115/142) had HRR wild-type status. A subgroup analysis by HRR aberration status, albeit not powered to detect a difference between subgroups, showed a similar rPFS benefit with the abi+ola combination compared with abi alone in the HRR mutant (HR 0.74), HRR partially characterized (HR 0.67), or HRR wild-type (HR 0.52) patients. This suggests that this synergy exists independent of a deleterious HRR alteration.

As expected, abi+ola combination had higher AE rates, with 93% (66/71) of patients experiencing an AE compared with 80% (57/71) in the abi-alone arm. Most of these events were grade 1–2, but rates of grade 3 or worse AEs were also higher in the abi+ola combination (54%; 38/71) compared with abi alone (28%; 20/71). An important AE of note was the 6% (4/71) rate of myocardial infarction in the abi+ola combination arm compared with 0% (0/71) in the abi-alone arm.

Data from the RAMP phase Ib trial of rucaparib and enzalutamide were also recently reported [73,74]. Patients with mCRPC who had received up to two lines of AR-directed therapy and up to two lines of chemotherapy were treated with a 1-week run-in of rucaparib monotherapy (600 mg two times per day) followed by rucaparib (600 mg two times a day) plus enzalutamide (160 mg daily) in continuous 28-day cycles. This combination showed an acceptable safety profile with no DLTs in six evaluable patients and no evidence of synergistic or unexpected treatment-related adverse events. Only one patient had an HRR gene aberration identified (subclonal CHEK2 alteration with <1% allele frequency)—this patient did not have a clinical response (as assessed by any PSA decline from baseline). No BRCA1/2 or PALB2 alterations were identified. There was early evidence of efficacy with 4/8 (50%) patients with a confirmed PSA response (reduction of ≥50% from baseline), and the single patient with measurable disease achieved a confirmed complete radiographic response.

### 5.2. Ongoing Clinical Trials of AR and PARP Combination Therapies—Highlights and Key Considerations for the Near Future

There are currently four major clinical trials that are evaluating NAA and PARP inhibitor combinations in mCRPC (Table 2). The key differences in their patient selection criteria, primary endpoints, and additional items of interest are outlined below.

The confirmatory trial of abiraterone and olaparib combination is ongoing (PROPEL, Clinicaltrials.gov ID: NCT03732820) [77], and the top-line data are expected in early 2022. This double-blind, placebo-controlled, randomized phase 3 trial will enroll 720 first-line mCRPC patients, who will be randomized in a 1:1 fashion to abiraterone plus olaparib or placebo. Patients are not eligible if they have received NAA therapy. The primary endpoint of this study is rPFS, and a key secondary endpoint is OS. This study may usher in an era of combination therapies in mCRPC, albeit only for NAA-naïve patients. The safety data from this study will allow greater insights into the cardiovascular AEs observed in the phase II study of abiraterone and olaparib, as discussed above. 

The CASPAR trial (A031902; Clinicaltrials.gov ID: NCT04455750) is investigating the clinical efficacy of enzalutamide and rucaparib combination [75,76]. This randomized, placebo-controlled, phase 3 trial will enroll 1002 first-line mCRPC patients, who will be randomized in a 1:1 fashion to enzalutamide plus rucaparib or placebo. It is currently enrolling patients and is expected to show preliminary results in 2023. Two key strengths of the CASPAR trial are (1) it addresses the changing treatment paradigm by allowing patients to have received NAA therapy for non-mCRPC disease settings, and (2) it is the only NAA and PARP combination trial that has co-primary endpoints of OS and rPFS, with an intent of answering the question of whether concurrent versus sequential PARP inhibitor therapy is more effective.

The MAGNITUDE trial (Clinicaltrials.gov ID: NCT03748641) of abiraterone and niraparib is ongoing [78]. In this randomized phase 3 trial, patients with first-line mCRPC will be treated in two cohorts based on DDR mutation status. The first cohort will comprise 400 patients with DDR mutations who will be randomized 1:1 to abiraterone plus niraparib or placebo. The second cohort will comprise 600 patients without DDR mutations who will be randomized 1:1 to abiraterone plus niraparib or placebo. The primary endpoint of this study is also rPFS. Interestingly, the study design has similar hazard ratio estimates for benefits of the combination in DDR mutant (92% power to detect HR ≤ 0.65) and DDR-wild type (94% power to detect HR ≤ 0.67) cohorts, suggesting that the study team has confidence in the underlying biology of synthetic lethality of the combination in patients without DDR mutations. 

Finally, the TALAPRO-2 study (Clinicaltrials.gov ID: NCT03395197) of enzalutamide and talazoparib is ongoing [80]. This randomized, phase 3 trial will enroll 1037 NAA-naïve patients with mCRPC to enzalutamide plus talazoparib or placebo. The first part (single-arm, dose-finding cohort) has completed enrollment of 19 patients, and part 2 (double-arm, placebo-controlled cohort) is now enrolling.

## 6. Discussion

Powered by strong underlying biology and mechanistic rationale, the ongoing clinical trials of NAA and PARP combinations are likely to create therapeutic breakthroughs. Important issues that merit close inspection include (1) whether an improvement in rPFS alone is sufficient justification for upfront combination therapy, or at least some of these patients derive similar benefit from sequencing (NAA and then PARP upon progression); (2) relevance and validity of trial results in the changing treatment landscape with increasing usage of NAA in pre-mCRPC settings; (3) differences in AE profiles of various combinations; (4) whether combination treatment is required until disease progression, or can a less-intensive approach (pulsed, intermittent PARP inhibition) yield similar benefits while improving treatment tolerability; and (5) biomarkers of response and resistance. If successful, these efforts are likely to spark a flurry of clinical trials in earlier disease settings as well. 

### Moving it Upstream—Does NAA and PARP Co-Inhibition Have a Role in Earlier Disease Settings?

Two unique aspects of currently available treatments have hampered treatment intensification in earlier disease states—ADT by itself has good performance and provides benefits in a plurality of patients, and ADT is also tolerated very well, which makes it harder to justify the added treatment AEs and financial burden associated with treatment intensification. Docetaxel, abiraterone, enzalutamide, and apalutamide have shown benefits and have been included in NCCN guidelines for mHSPC over the past 5 years. Yet, the adoption rates of these therapies have been sluggish, even for patients with high-volume disease who generally have symptomatic disease and significantly lower magnitude of benefit with ADT alone. This is in contrast to anecdotal evidence from other malignancies where even chemoimmunotherapy triplet therapies (e.g., lung cancer) have seen a surge in adoption shortly after their FDA approval. 

A PARP-based combination is likely to face similar challenges in mHSPC. There is the issue of optimal patient selection because not all patients with mHSPC will need or benefit from treatment intensification. Even if we disregard this issue, the added treatment-related AEs and financial burden are expected to be significant barriers to the adoption and widespread usage of NAA and PARP inhibitor combinations in mHSPC. 

Another potentially “high-value” niche for these combinations exists in the localized/locally advanced prostate cancer setting. RT + ADT is the standard of care for unfavorable intermediate or high-risk localized disease and locally advanced disease based on the unequivocal improvement in biochemical and distant metastatic recurrence-free survival and overall survival [81]. However, there remains an opportunity for improvement in clinical outcomes given the recurrence rate of approximately 25% at 5 years despite the use of RT + ADT [82]. Recently, the addition of abiraterone with or without enzalutamide to the RT + ADT combination in the STAMPEDE trial was shown to improve the overall survival for men with very-high risk localized or locally advanced prostate cancer [83]. The 6-year metastasis-free survival improved from 69% to 82%. However, this still leaves almost 20% of men experiencing metastasis in the first 6 years after treatment.

A key trial in this space is the randomized, phase II trial of abiraterone, apalutamide, niraparib, ADT, and radiation for patients with high-risk localized or locally advanced prostate cancer (ClinicalTrials.gov ID: NCT04947254). In this ambitious trial, patients will receive neoadjuvant apalutamide and ADT for up to 3 cycles of 28 days each, followed by radiation, apalutamide, and ADT. Patients with a favorable response to this regimen will proceed with adjuvant apalutamide and ADT for up to 12 cycles. Patients without a favorable response to the initial regimen will be randomized to either adjuvant apalutamide and ADT for up to 12 cycles of treatment intensification with abiraterone, niraparib, and ADT for up to 12 cycles. Co-primary endpoints are rPFS and PSA-PFS.

Other noteworthy PARP inhibitor combination trials include the NADIR phase 1/2 trial, evaluating a combination of niraparib and ADT concurrently with RT in very-high-risk prostate cancer (ClinicalTrials.gov ID: NCT04037254); the ASCLEPlus phase 1/2 trial, evaluating abiraterone, niraparib, and ADT with stereotactic body RT to the prostate gland (ClinicalTrials.gov ID: NCT04194554); and the FAALCON phase 2 trial, evaluating “pulsed” systemic therapy using abiraterone and ADT for 6 months, olaparib for 5 months, and RT to all (≤5) oligometastatic sites of hormone-sensitive prostate cancer (ClinicalTrials.gov ID: NCT04748042).

With the proliferation of genomic classifiers and clinical decision-making tools (e.g., CAPRA score) to guide individualized risk assessment and treatment selection and the possibility of cure and the finite duration of therapy in curative and adjuvant settings, this niche is likely to remain an active area of research and clinical development of NAA and PARP inhibitor combinations. 

## 7. Conclusions

The discovery of induced synthetic lethality using co-inhibition of AR and PARP has opened the doors for investigation of this combination approach as a new treatment option for advanced prostate cancer. This comes at a time where existing treatment options have found increasing use in earlier disease settings, and our understanding of de novo or early onset NAA resistance has increased. It is clear that a subset of patients with advanced prostate cancer has an urgent, unaddressed need for treatment intensification. Several promising clinical trials are underway to evaluate the role of NAA and PARP combinations and may overhaul treatment options for mCRPC patients in the near future.

## Figures and Tables

**Table 1:** A summary of key prospective and retrospective studies evaluating sequencing of abiraterone and enzalutamide in mCRPC.

Study	Study Type	Sequence (n)	Median PFS	Median PSA-PFS **	Median OS
Khalaf et al. [10]	Prospective, randomized	AAP → ENZ (101)	-	19·3 months (95% CI 16·0–30·5)	28.8 months (95% CI 25.4–NR)
		ENZ → AAP (101)	-	15.2 months (95% CI 11.9–19.8) (*p* = 0.036)	24.7 months (95% CI 18.8–34.0) (*p* = 0.23)
Terada et al. [11] ^##^	Retrospective	AAP → ENZ (113)	-	15.0 months (95% CI 12.6–16.3)	30.2 months (95% CI 25.0–NR)
		ENZ → AAP (85)	-	9.7 months (95% CI 7.7–11.8) (*p* < 0.001)	29.5 months (95% CI 24.4–NR) (*p* = 0.599)
Mori et al. [12]	Retrospective	AAP → ENZ (23)	NR	9 months	Not statistically different (*p* = 0.62)
		ENZ → AAP (46)	11 months (*p* = 0.043)	7 months (*p* = 0.049)	
Maughan et al. [11]	Retrospective	AAP → ENZ (65)	19.5 months (95% CI 15.5–22.3)	17.5 months (95% CI 14.0–19.5)	33.3 months (95% CI 2.4–NR)
		ENZ → AAP (16)	13.0 months (95% CI 10.3–21.2)	12.3 months (95% CI 8.9–20.5)	29.9 months (95% CI 18.8–NR)

**—For Khalaf et al., the primary endpoint was time to second PSA progression, and these data are reported under median PSA-PFS column; N—number of patients; PFS—progression-free survival; OS—overall survival; AAP—abiraterone acetate and prednisone; ENZ—enzalutamide; NR - not reached; CI—confidence interval; ^##^—For Terada et al., PSA-PFS and OS data have been converted from days to months for consistency.

**Table 2:** Contemporary clinical trials evaluating NAA and PARP inhibitor combinations.

Study	Study Design	Arms (n)	Patient Selection	Primary Endpoint
CASPAR/ A031902 [75,76]	Phase 3, randomized, placebo-controlled, double-blinded	Enzalutamide + Rucaparib (496) Enzalutamide + Placebo (496)	Prior NAA allowed for mHSPC and nmCRPC Prior docetaxel allowed for mHSPC HRR mutation not required	Radiographic PFS and overall survival
PROPEL [77]	Phase 3, randomized, placebo-controlled, double-blinded	Abiraterone + Olaparib (360) Abiraterone + Placebo (360)	Prior NAA NOT allowed Prior docetaxel allowed for mHSPC HRR mutation not required	Radiographic PFS
MAGNITUDE [78]	Phase 3, randomized, placebo-controlled, double-blinded	**HRR-mutant cohort** Enzalutamide + Rucaparib (200) Enzalutamide + Placebo (200)	Prior NAA NOT allowed for mHSPC and nmCRPC Prior docetaxel allowed for mHSPC HRR mutation not required	Radiographic PFS in HRR-mutant patients
		**HRR-wt cohort** Enzalutamide + Rucaparib (300) Enzalutamide + Placebo (300)	Prior NAA NOT allowed for mHSPC and nmCRPC Prior docetaxel allowed for mHSPC HRR mutation not required	Radiographic PFS in HRR-wt patients
TALAPRO-2 [79]	Phase 3, randomized, placebo-controlled, double-blinded	Enzalutamide + Talazoparib (509) Enzalutamide + Placebo (509)	Prior abiraterone allowed (no novel AR inhibitors) for mHSPC and nmCRPC Prior docetaxel allowed for mHSPC HRR mutation not required	Radiographic PFS

n—number of patients in the arm; NAA—novel antiandrogen; mHSPC—metastatic hormone-sensitive prostate cancer; nmCRPC—non-metastatic castration-resistant prostate cancer; HRR—homologous recombination repair gene; wt—wild-type (non-mutated); PFS—progression-free survival.
